# Urinary Concentrations of Organophosphate Flame-Retardant Metabolites in the US Population

**DOI:** 10.1001/jamanetworkopen.2024.35484

**Published:** 2024-09-25

**Authors:** Yu-Song Huang, Hui-Zhong Shi, Xi Huang, Yi-Ming Pan, Yu-Chen Wang, Zi-Jun Gao, Pei-Yao Jiang, Wen-Yi Yang

**Affiliations:** 1 Department of Cardiology, Shanghai General Hospital, Shanghai Jiao Tong University School of Medicine, Shanghai, China; 2 Department of Hematology, Shanghai General Hospital, Shanghai Jiao Tong University School of Medicine, Shanghai, China; 3 Department of Critical Care Medicine, Ren Ji Hospital, Shanghai Jiao Tong University School of Medicine, Shanghai, China; 4 Institute of Blood Transfusion, Chinese Academy of Medical Sciences and Peking Union Medical College, Sichuan, China

## Abstract

**Importance:**

Organophosphate flame retardants (OPFRs) are an important group of pollutants associated with endocrine disorders, cancer, and nephrotoxicity. However, temporal trends in OPFR metabolite concentrations remain understudied.

**Objectives:**

To examine changes in urinary concentrations of OPFR metabolites among US children, youths, and adults from 2011 to 2020, and to evaluate whether sociodemographic factors were associated with variations in temporal trends.

**Design, Setting, and Participants:**

This population-based cross-sectional study used data from 4 US National Health and Nutrition Examination Survey (NHANES) cycles (2011-2012, 2013-2014, 2015-2016, and 2017-2020 [to March 2020 before the COVID-19 pandemic]). The study included children and youths (aged 6-19 years) and adults (aged ≥20 years) with valid urinary concentrations of the following OPFR metabolites: bis(2-chloroethyl) phosphate (BCEtP), bis(1-chloro-2-propyl) phosphate (BCPP), diphenyl phosphate (DPhP), and dibutyl phosphate (DBuP). Data analysis was performed between February and May 2024.

**Exposures:**

Calendar year and key sociodemographic subgroups (age, race and ethnicity, sex, educational attainment, and poverty-to-income ratio).

**Main Outcomes and Measures:**

The main outcome was urinary concentrations of OPFR metabolites among children, youths, and adults. Survey-weighted linear regression models were applied to estimate trends.

**Results:**

The study population of 10 549 NHANES participants included 3154 children and youths (mean [SE] age, 12.5 [0.1] years; 51.2% were male) and 7395 adults (mean [SE] age, 47.8 [0.4] years; 52.0% were women). Among children and youths, mean (95% CI) BCEtP concentrations decreased from 0.68 (0.60-0.77) μg/L in 2011-2012 to 0.41 (0.37-0.45) μg/L in 2017-2020 (*P* for trend < .001). Among adults, mean (95% CI) BCEtP concentrations decreased from 0.43 (0.37-0.50) μg/L in 2011-2012 to 0.29 (0.27-0.33) μg/L in 2017-2020 (*P* for trend < .001), and mean BCPP concentrations decreased from 0.15 (0.14-0.17) μg/L to 0.13 (0.12-0.14) μg/L (*P* for trend = .002). Parent level of educational attainment was associated with concentrations of BCPP and BCEtP among children and youths; however, no significant differences among adults were observed.

**Conclusions and Relevance:**

This study identified variations in temporal trends in urinary concentrations of OPFR metabolites among the US population from 2011 to 2020. In addition, substantial disparities in exposure levels persisted among children with different levels of parent educational attainment. These findings suggest that policy makers should consider socioeconomic factors to further reduce OPFR exposure and promote equity, ensuring a safe living environment for all individuals.

## Key Points

**Question:**

How did urinary concentrations of organophosphate flame-retardant (OPFR) metabolites among the US population change from 2011 to 2020, and were sociodemographic factors associated with higher risk for OPFR exposure?

**Findings:**

In this cross-sectional study of 10 549 US National Health and Nutrition Examination Survey participants aged 6 years or older, bis(2-chloroethyl) phosphate (BCEtP) concentrations decreased among children and youths and both bis(1-chloro-2-propyl) phosphate and BCEtP concentrations decreased among adults, with slight variations over the study period. Substantial disparities in exposure levels persisted among children with different levels of parent educational attainment.

**Meaning:**

These findings suggest that OPFR exposure remains a critical public health issue and that the implementation of effective, targeted regulatory strategies is needed.

## Teaser

This cross-sectional study uses US National Health and Nutrition Examination Survey data to examine temporal and sociodemographic trends in urinary concentrations of organophosphate flame-retardant metabolites among children, youths, and adults from 2011 to 2020.

## Introduction

In recent years, organophosphorus flame retardants (OPFRs) have been used widely in various consumer products, including electronics, furniture, and textiles, due to their effective flame-retardant properties.^1^ However, growing evidence suggests that OPFRs pose health risks, including endocrine disruption, neurodevelopmental issues, and other adverse outcomes.^2,3,4^ Because these chemicals are incorporated into products rather than covalently bound, they can leach into the environment, resulting in human exposure mainly through inhalation, ingestion, and skin contact.^5,6^

Biomonitoring studies have detected OPFR metabolites in human urine, indicating widespread exposure in the general population.^7,8^ Bis(2-chloroethyl) phosphate (BCEtP), bis(1-chloro-2-propyl) phosphate (BCPP), diphenyl phosphate (DPhP), and dibutyl phosphate (DBuP) are OPFR metabolites that can be quantified in urine.^9^ Previous studies have shown that concentrations of OPFRs and OPFR metabolites increased between 2002 and 2015, but the study populations were not representative of the general population and only focused on exposure levels of 2 flame retardants up to 2015.^10^ There are also studies that have analyzed temporal trends for various OPFR metabolites, but they were limited to pregnant women.^11,12^ Although older or smaller-scale studies have analyzed trends in OPFR metabolites, recent nationally representative estimates among US population subgroups are lacking.^10,11,12^

In this study, we used US National Health and Nutrition Examination Survey (NHANES) data for 2011 to 2020 to analyze trends in urinary concentrations of 4 OPFR metabolites among the US population. Understanding of these trends is critical for conducting public health surveillance, guiding future regulatory policies, and developing targeted interventions to mitigate exposure risks. We focused on DPhP, BCPP, BCEtP, and DBuP, which correspond to the parent chemicals triphenyl phosphate (TPhP), tris(1-chloro-2-propyl) phosphate (TCPP), tris(2-chloroethyl) phosphate (TCEP), and tributyl phosphate (TBP), respectively. We also sought to identify sociodemographic groups that may be at heightened risk for exposure, thereby highlighting potential disparities in exposure levels. We hypothesized that individuals with higher household incomes or with higher educational attainment would be more likely to have lower and decreasing concentrations of these metabolites over the study period.

## Methods

The Shanghai General Hospital Ethics Committee deemed this cross-sectional study exempt from ethical review and informed consent because publicly available and deidentified NHANES data were used. For NHANES cycles, researchers recruit participants using established consent protocols approved by the National Center for Health Statistics Institutional Review Board.^13^ Details of the data collection procedures, study design, and protocol were published previously.^14^ This study followed the Strengthening the Reporting of Observational Studies in Epidemiology (STROBE) reporting guideline.

### Data Source

We used data from the NHANES, which is conducted biennially by the US Centers for Disease Control and Prevention (CDC) for public health surveillance.^15^ The CDC suspended NHANES data collection in March 2020 due to the COVID-19 pandemic. Therefore, we used data from NHANES cycles from 2011 to March 2020 (prepandemic). Because data collection for the 2019-2020 NHANES cycle was not completed and the collected data were not nationally representative, we used 2017-2020 prepandemic NHANES data to ensure nationally representative estimates.^16^ Additionally, we adjusted the weights of the prepandemic period based on official recommendations to minimize potential bias.

### Study Population

We included participants aged 6 years or older who completed the NHANES household interview and provided stored urine specimens between 2011 and March 2020. The NHANES investigators began collecting race and ethnicity data for Asian participants in 2011, with estimates included from 2011 onward.^16^ Race and ethnicity were self-reported by study participants based on fixed category responses, which included Hispanic (Mexican American or other Hispanic ethnicity), non-Hispanic Asian (hereinafter, Asian), non-Hispanic Black (hereinafter, Black), non-Hispanic White (hereinafter, White), and other race or ethnicity (American Indian or Alaska Native, Native Hawaiian or Other Pacific Islander, multiple races, or individuals who did not identify as Asian, Black, Hispanic, or White). These categories were included in the analyses to determine whether urinary concentrations of OPFR metabolites varied across different racial and ethnic subgroups. Participant sex was defined based on self-report. Income was assessed using the poverty-to-income ratio (PIR), calculated as family income divided by the poverty guidelines specific to the survey year. Low income was defined as a PIR of less than 1.30, middle income as a PIR between 1.30 and 3.49, and high income as a PIR of 3.50 or greater.

### Urinary Concentrations of OPFR Metabolites

Urine specimens were collected, frozen, and securely packed in dry ice and then shipped in cryovials to the National Center for Environmental Health, where they were stored at controlled temperatures (≤−20 °C) until analysis.^17^ Urinary OPFR metabolites were analyzed using automated offline solid-phase extraction and reversed-phase high-performance liquid chromatography separation, followed by isotope dilution−electrospray ionization tandem mass spectrometry. The laboratory methodology was reported previously.^18^ We focused on 4 OPFR metabolites (DPhP, BCPP, BCEtP, and DBuP) for subsequent statistical analysis because these were consistently available across all cycles in the 2011-2020 prepandemic NHANES data.

### Statistical Analysis

The analysis used NHANES special sampling weights for OPFR metabolites, accounting for sampling, stratification, and clustering to ensure nationally representative estimates.^19,20^ The 2017-2020 prepandemic NHANES data file spans 3.2 years, whereas other NHANES data files cover 2-year periods. Due to this difference, survey weights were adjusted when combining the 2017- 2020 prepandemic NHANES data files with other 2-year cycles to reflect the longer period and larger population. New multicycle sample weights were calculated based on the sample weights of the combined survey cycles (2011-2012, 2013-2014, 2015-2016, and 2017-2020, totaling 9.2 years) using the following formulas^16^: (1) Weight = Weight × (2/9.2), if the cycle was 2011-2012, 2013-2014, or 2015-2016; and (2) Weight = Weight × (3.2/9), if the cycle was 2017-2020.

In this study, we describe the baseline characteristics of individuals, categorized as either children and youths or adults. We report means (SEs) for continuous variables and percentages for categorical variables. Because the OPFR metabolites had a skewed distribution, we calculated geometric means (hereinafter, means) for these concentrations. Confidence intervals were calculated using Taylor series linearization. To estimate trends, a survey-weighted linear regression model was applied, treating the survey cycle as a continuous variable.^21^ Results are reported for 4 NHANES cycles (2011-2012, 2013-2014, 2015-2016, and 2017-2020) to ensure sufficient sample size. Potential differences in trends among subpopulations were examined using survey-weighted Wald *F* statistics. This method assessed the interaction between survey cycles and indicators of subpopulations (age, race and ethnicity, sex, educational attainment, and PIR).^22^

Statistical analysis was performed with R, version 4.1.3 (R Project for Statistical Computing), and the NHANES design was accounted for using the survey package for R.^23^ Statistical significance was determined with an α of ≤.05 (2-tailed). Data analysis was performed between February and May 2024.

## Results

### Participant Characteristics

The study population of 10 549 participants included 3154 children and youths (aged 6-19 years) and 7395 adults (aged ≥20 years). Baseline characteristics of participants are presented in Table 1. Children and youths had a mean (SE) age of 12.5 (0.1) years; 51.2% were male and 48.8% were female. A total of 4.7% of children and youths were Asian, 14.1% were Black, 23.5% were Hispanic, 52.7% were White, and 5.0% were of other race or ethnicity. Adults had a mean (SE) age of 47.8 (0.4) years; 48.0% were men and 52.0% were women. In terms of race and ethnicity, 5.5% of adults were Asian, 11.5% were Black, 15.1% were Hispanic, 64.3% were White, and 3.6% were of other race or ethnicity.

**Table 1.** Baseline Characteristics of Participants Aged 6 Years or Older, 2011-2020 NHANES Cycles^a^

Characteristic	Children and youths (aged 6-19 y)				Adults (aged ≥20 y)			
	2011-2012 (n = 619)^b^	2013-2014 (n = 686)	2015-2016 (n = 631)	2017-2020 (n = 1218)	2011-2012 (n = 1435)	2013-2014 (n = 1631)	2015-2016 (n = 1447)	2017-2020 (n = 2882)
Age, mean (SE), y	12.4 (0.2)	12.4 (0.2)	12.4 (0.2)	12.6 (0.2)	47.4 (0.9)	47.5 (0.5)	47.8 (0.9)	48.3 (0.6)
Sex^c^								
Male	320 (50.8)	369 (51.5)	334 (51.2)	634 (51.1)	708 (47.9)	788 (48.0)	690 (48.1)	1434 (48.1)
Female	299 (49.2)	317 (48.5)	297 (48.8)	584 (48.9)	727 (52.1)	843 (52.0)	757 (51.9)	1448 (51.9)
Race and ethnicity								
Asian	78 (4.7)	59 (4.5)	56 (5.0)	98 (4.8)	191 (5.2)	179 (5.3)	146 (5.6)	341 (6.0)
Black	174 (15.0)	164 (13.7)	125 (14.1)	331 (13.7)	341 (11.6)	302 (11.4)	304 (11.3)	790 (11.5)
Hispanic	186 (22.1)	225 (23.0)	249 (23.8)	318 (24.9)	297 (14.2)	364 (14.7)	444 (15.3)	624 (16.0)
White	149 (54.7)	194 (52.5)	173 (53.2)	363 (50.5)	567 (65.8)	737 (66.0)	486 (63.1)	998 (62.5)
Other^d^	32 (3.4)	44 (6.3)	28 (4.0)	108 (6.0)	39 (3.1)	49 (2.6)	67 (4.7)	129 (4.0)
Education^e^								
Some high school or less	151 (22.4)	173 (18.7)	173 (20.5)	NA	328 (16.3)	320 (14.1)	328 (13.2)	557 (10.8)
High school graduate	127 (20.2)	140 (19.0)	126 (20.3)	NA	306 (19.7)	361 (20.6)	322 (21.0)	667 (26.8)
Some college	165 (26.7)	219 (33.5)	192 (33.5)	NA	439 (32.8)	528 (34.2)	458 (34.5)	959 (30.8)
College degree or greater	151 (27.4)	143 (27.1)	118 (22.9)	NA	362 (31.2)	420 (30.9)	337 (31.3)	694 (31.6)
Missing	25 (3.3)	11 (1.7)	22 (2.8)	NA	0	2 (0.07)	2 (0.03)	5 (0.07)
Family PIR								
<1.30	267 (33.5)	297 (33.7)	245 (28.0)	444 (27.1)	484 (23.3)	502 (22.2)	412 (18.4)	693 (16.5)
1.30-3.49	192 (36.5)	202 (32.2)	219 (38.0)	392 (30.8)	446 (31.9)	553 (33.5)	509 (32.0)	967 (29.5)
≥3.50	123 (25.3)	154 (30.4)	115 (27.8)	242 (31.1)	411 (39.9)	459 (38.9)	392 (42.5)	808 (42.6)
Missing	37 (4.7)	33 (3.6)	52 (6.2)	140 (11.0)	94 (4.9)	117 (5.4)	134 (7.1)	414 (11.4)

Abbreviations: NA, not appliable; NHANES, US National Health and Nutrition Examination Survey; PIR, poverty-to-income ratio.

^a^ Unless indicated otherwise, data are presented as No. (%) of participants. Data are presented incorporating sample weights and adjusted for clusters and strata of the complex sample design of the 2011-2020 NHANES cycles.

^b^ Unweighted sample sizes are presented for n values in the column headings.

^c^ Because all numbers were rounded, percentages may not sum to 100%.

^d^ Includes American Indian or Alaska Native, Native Hawaiian or Other Pacific Islander, multiple races or ethnicities, and not identifying as Asian, Black, Hispanic, or White.

^e^ Refers to parent level of educational attainment.

### Trends in Urinary Concentrations of OPFR Metabolites Among Children, Youths, and Adults

Among children and youths, mean (95% CI) BCEtP concentrations decreased from 0.68 (0.60-0.77) μg/L in 2011-2012 to 0.41 (0.37-0.45) μg/L in 2017-2020 (*P* for trend < .001; Figure A and Table 2). Mean (95% CI) concentrations of DPhP (1.56 [1.38-1.75] μg/L vs 1.51 [1.41-1.62] μg/L; *P* for trend = .89), BCPP (0.17 [0.15-0.18] μg/L vs 0.16 [0.14-0.18] μg/L; *P* for trend = .21), and DBuP (0.12 [0.10-0.14] μg/L vs 0.15 [0.14-0.17] μg/L; *P* for trend = .62) remained stable for the same period (Figure A and Table 2).

**Figure.**
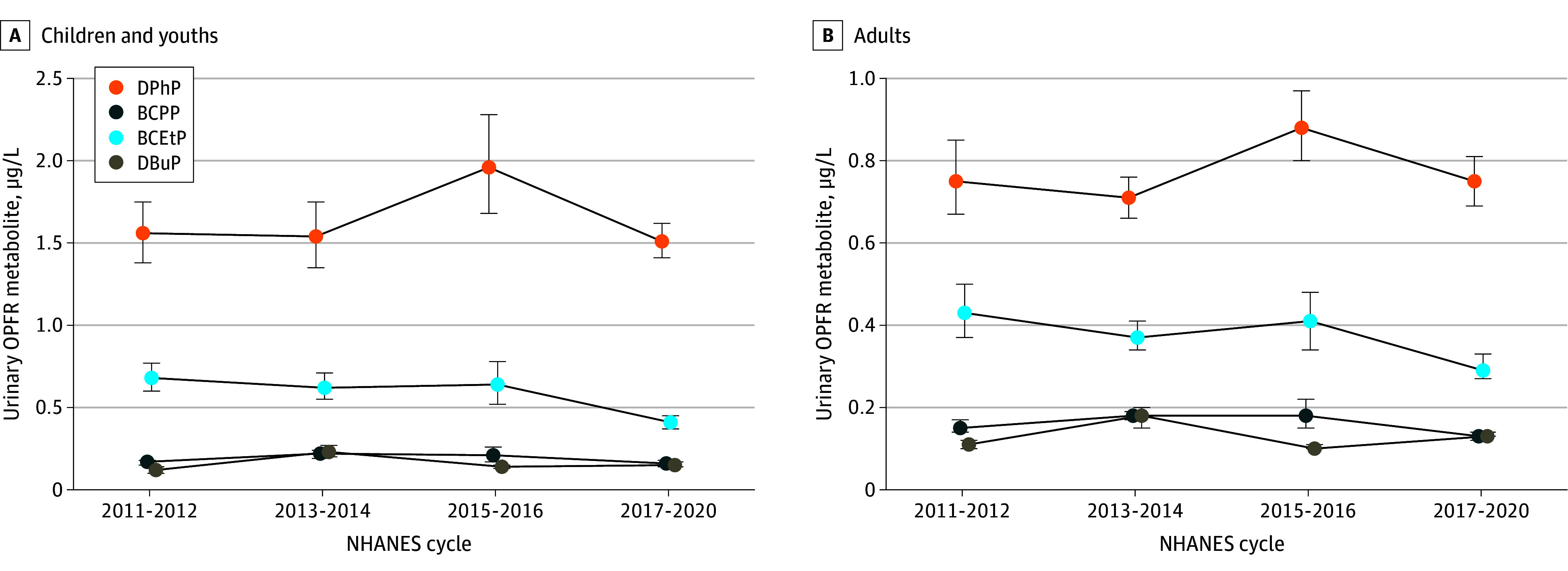
Urinary Concentrations of Organophosphate Flame-Retardant (OPFR) Metabolites Among US Children, Youths, and Adults, 2011 to 2020 A and B, Urinary concentrations of OPFR metabolites among US children and youths (A) and adults (B). Data are presented incorporating sample weights and adjusted for clusters and strata of the complex sample design of the National Health and Nutrition Examination Survey (NHANES) for 4 cycles (2011-2012, 2013-2014, 2015-2016, and 2017-2020). BCEtP indicates bis(2-chloroethyl) phosphate; BCPP, bis(1-chloro-2-propyl) phosphate; DBuP, dibutyl phosphate; DPhP, diphenyl phosphate.

**Table 2.** Urinary Concentrations of OPFR Metabolites Among Children, Youths, and Adults, 2011-2020 NHANES Cycles^a^

OPFR metabolite	Children and youths (aged 6-19 y)					Adults (aged ≥20 y)				
	Geometric mean (95% CI)				*P* value for trend^b^	Geometric mean (95% CI)				*P* value for trend
	2011-2012	2013-2014	2015-2016	2017-2020		2011-2012	2013-2014	2015-2016	2017-2020	
DPhP	1.56 (1.38-1.75)	1.54 (1.35-1.75)	1.96 (1.68-2.28)	1.51 (1.41-1.62)	.89	0.75 (0.67-0.85)	0.71 (0.66-0.76)	0.88 (0.80-0.97)	0.75 (0.69-0.81)	.58
BCPP	0.17 (0.15-0.18)	0.22 (0.19-0.24)	0.21 (0.17-0.26)	0.16 (0.14-0.18)	.21	0.15 (0.14-0.17)	0.18 (0.17-0.19)	0.18 (0.15-0.22)	0.13 (0.12-0.14)	.002
BCEtP	0.68 (0.60-0.77)	0.62 (0.55-0.71)	0.64 (0.52-0.78)	0.41 (0.37-0.45)	<.001	0.43 (0.37-0.50)	0.37 (0.34-0.41)	0.41 (0.34-0.48)	0.29 (0.27-0.33)	<.001
DBuP	0.12 (0.10-0.14)	0.23 (0.20-0.27)	0.14 (0.13-0.15)	0.15 (0.14-0.17)	.62	0.11 (0.10-0.12)	0.18 (0.15-0.20)	0.10 (0.10-0.11)	0.13 (0.13-0.14)	.91

Abbreviations: BCEtP, bis(2-chloroethyl) phosphate; BCPP, bis(1-chloro-2-propyl) phosphate; DBuP, dibutyl phosphate; DPhP, diphenyl phosphate; NHANES, US National Health and Nutrition Examination Survey; OPFR, organophosphate flame retardant.

^a^ Unless indicated otherwise, data are presented as geometric means (95% CIs) due to the skewed distribution. Estimates were adjusted for NHANES survey weight.

^b^ Tested across cycles to evaluate the monotonic trend across the whole period.

Among adults, mean (95% CI) BCPP concentrations decreased from 0.15 (0.14-0.17) μg/L in 2011-2012 to 0.13 (0.12-0.14) μg/L in 2017-2020 (*P* for trend = .002; Figure B and Table 2). Mean (95% CI) BCEtP concentrations decreased from 0.43 (0.37-0.50) μg/L in 2011-2012 to 0.29 (0.27-0.33) μg/L in 2017-2020 (*P* for trend < .001; Figure B and Table 2). Mean (95% CI) DPhP concentrations (0.75 [0.67-0.85] μg/L and 0.75 [0.69-0.81] μg/L; *P* for trend = .58) and DBuP (0.11 [0.10-0.12] μg/L and 0.13 [0.13-0.14] μg/L; *P* for trend = .91) remained stable for the same period (Figure B and Table 2).

### Trends in Urinary Concentrations of OPFR Metabolites by Population Subgroups

#### DPhP Concentrations

Changes in DPhP concentrations were similar across most population subgroups of children and youths (eFigures 1, 3, and 5 and eTable 1 in Supplement 1). However, trends differed significantly by sex over time (*P* for interaction < .001). Mean (95% CI) DPhP concentrations decreased by approximately 0.7 (0.4-1.0) μg/L among female individuals and increased by approximately 0.4 (0.2-0.7) μg/L for male individuals (both *P* for trend < .001; eFigure 2 and eTable 1 in Supplement 1).

Mean DPhP concentrations remained stable in households in which the parent level of educational attainment was less than a college degree (eFigure 4A-C and eTable 1 in Supplement 1). However, mean (95% CI) DPhP concentrations increased in households with at least 1 parent with a college degree or higher (1.17 [0.89-1.53] μg/L in 2011-2012 vs 1.99 [1.66-2.39] μg/L in 2015-2016; *P* for trend = .003; eFigure 4D and eTable 1 in Supplement 1).

From 2011 to 2020, DPhP concentrations among adults remained stable and were generally similar across most subgroups, except for age (eFigure 6 and eTable 2 in Supplement 1).

#### BCPP Concentrations

Changes in BCPP concentrations were statistically significant among some subgroups, whereas they remained stable among others (eFigures 1-10 and eTables 1 and 2 in Supplement 1). Among children and youths of various racial and ethnic groups, mean (95% CI) BCPP concentrations decreased among Black individuals (0.19 [0.16-0.22] μg/L in 2011-2012 vs 0.15 [0.13-0.17] μg/L in 2017-2020; *P* for trend = .03) and Hispanic individuals (0.18 [0.15-0.20] μg/L vs 0.14 [0.12-0.16] μg/L; *P* for trend = .008), whereas concentrations remained stable for Asian individuals, White individuals, and individuals of other races or ethnicities (eFigure 3 and eTable 1 in Supplement 1).

Across different levels of parent educational attainment, mean (95% CI) BCPP concentrations increased significantly among children and youths with parents who had a college degree or greater (0.15 [0.13-0.17] μg/L in 2011-2012 vs 0.24 [0.18-0.33] μg/L in 2015-2016; *P* for trend = .007) but did not change significantly for children and youths whose parents did not graduate from college (*P* for interaction = .02; eFigure 4 and eTable 1 in Supplement 1).

Mean (95% CI) BCPP concentrations decreased significantly among younger adults (aged 20-39 years; 0.17 [0.14-0.20] μg/L in 2011-2012 vs 0.13 [0.12-0.15] μg/L in 2017-2020; *P* for trend = .002) but did not change significantly for older adults (aged ≥40 years; eFigure 6 and eTable 2 in Supplement 1). In terms of sex, mean (95% CI) BCPP concentrations did not change significantly among men (0.15 [0.13-0.17] μg/L in 2011-2012 vs 0.14 [0.13-0.15] μg/L in 2017-2020; *P* for trend = .06) but decreased among women (0.15 [0.14-0.17] μg/L vs 0.13 [0.12-0.13] μg/L; *P* for trend < .001; eFigure 7 and eTable 2 in Supplement 1).

In terms of race and ethnicity, mean (95% CI) BCPP concentrations decreased significantly among Asian adults (0.17 [0.14-0.21] μg/L in 2011-2012 vs 0.13 [0.11-0.15] μg/L in 2017-2020; *P* for trend = .02), Black adults (0.18 [0.16-0.20] μg/L vs 0.13 [0.12-0.15] μg/L; *P* for trend < .001), Hispanic adults (0.15 [0.14-0.17] μg/L vs 0.13 [0.12-0.14] μg/L; *P* for trend = .009), and White adults (0.15 [0.13-0.17] μg/L vs 0.13 [0.12-0.14] μg/L; *P* for trend = .03). However, BCPP concentrations did not change significantly among adults of other races or ethnicities (eFigure 8 and eTable 2 in Supplement 1).

Mean (95% CI) BCPP concentrations decreased significantly for individuals with a college degree or higher (0.16 [0.13-0.19] μg/L in 2011-2012 vs 0.13 [0.12-0.14] μg/L in 2017-2020; *P* for trend = .007) but remained unchanged for those with lower levels of educational attainment (eFigure 9 and eTable 2 in Supplement 1). Mean (95% CI) BCPP concentrations were stable in low-income and middle-income households but decreased in high-income households (0.16 [0.14-0.18] μg/L in 2011-2020 vs 0.14 [0.13-0.15] μg/L in 2017-2020; *P* for trend = .01; eFigure 10 and eTable 2 in Supplement 1).

#### BCEtP Concentrations

Statistically significant decreases in BCEtP concentrations were observed among some subgroups, whereas they remained stable among others (eFigures 1-10 and eTables 1 and 2 in Supplement 1). Among children and youths, BCEtP concentrations decreased significantly across age, sex, and household income subgroups as well as in some racial and ethnic subgroups (Black, Hispanic, and White) (eTable 1 in Supplement 1). However, BCEtP concentrations remained stable in other subgroups (Asian and other races or ethnicities) (eFigure 3 and eTable 1 in Supplement 1).

Across different levels of parent educational attainment, BCEtP concentrations among children and youths did not show significant changes. However, an association was observed for the interaction between patterns of parent educational attainment and changes in BCEtP concentrations (eFigure 4 and eTable 1 in Supplement 1).

Among adults, BCEtP concentrations decreased significantly across several subgroups. For example, associations were observed between decreases in BCEtP concentrations and age (20-39 and 40-59 years), sex, education (parent educational attainment of some high school or lower, some college, and college degree or greater), and household income (low, middle, and high). Associations were also observed between decreases in BCEtP concentrations and some racial and ethnic subgroups (Asian, Black, Hispanic, and White). However, BCEtP concentrations remained stable in the other subgroups (eg, individuals aged ≥60 years, those of other races or ethnicities, and high school graduates; eFigures 6-10 and eTable 2 in Supplement 1).

#### DBuP Concentrations

 Across population subgroups of children and youths, DBuP concentrations were generally similar and stable (eTable 1 in Supplement 1). Among children whose parents had some high school education or less, mean (95% CI) DBuP concentrations increased from 0.12 (0.10-0.14) μg/L in 2011-2012 to 0.14 (0.13-0.16) μg/L in 2015-2016 (*P* for trend = .03; eFigure 4A and eTable 1 in Supplement 1).

Among adults, DBuP concentrations remained mostly stable across subgroups, with some exceptions. Associations between DBuP concentrations and sex, education, and household income were observed (eTable 2 in Supplement 1).

## Discussion

To our knowledge, this study is the first comprehensive analysis of temporal trends of 4 OPFR metabolites (DPhP, BCPP, BCEtP, and DBuP) in the US population after their widespread adoption as alternatives to polybrominated diphenyl ethers. We observed a decreasing trend in BCEtP concentrations among children and youths, with minor fluctuations over the study period (2011 to March 2020). Similarly, we observed a decreasing trend in both BCPP and BCEtP concentrations among adults, with slight variations over time.

Several federal and state legislative measures may partially explain the decrease in BCEtP and BCPP concentrations.^24,25,26,27,28,29,30,31^ Publications and reports have highlighted the risks of TCEP and TCPP in consumer products and the environment, increasing awareness of and decreasing purchases and use of items containing these chemicals.^32^ Additionally, both the production and import of TCEP have decreased in the US by approximately 99% since 2014. According to 2020 chemical data, no company reported the manufacture or import of TCEP in the US from 2016 to 2020.^32^ Moreover, production of TCPP decreased by approximately 96% from 2015 to 2019 in the US.^32,33^

In this study, DPhP concentrations peaked in the 2015-2016 NHANES cycle. This finding is consistent with a study by Hoffman et al,^12^ which suggested that DPhP exposures peaked in 2015 and may decline in the future. Our findings expand and confirm the predictions of Hoffman et al. Triphenyl phosphate is used not only in flame retardants but also in plasticizers and lubricants, leading to greater environmental dispersion.^34^ This chemical is also found in personal care products such as nail polish, perfume, cosmetics, suntan lotion, and hair spray, which are commonly used by women.^35^ This widespread use of TPhP may partly explain the higher urinary concentrations of DPhP in women compared with men. Although US production of TPhP decreased by approximately 93% from 2011 to 2019, DPhP is a metabolite of multiple compounds and is used in various products.^12,32^ Therefore, its presence in urine does not necessarily indicate exposure to TPhP alone.

In this study, urinary concentrations of DBuP among children, youths, and adults remained relatively stable from 2011 to 2020, with no significant trends observed. From 2011 to 2019, TBP production in the US increased by approximately 1.2-fold.^32^ Its biodegradability and lack of a clear regulatory ban may explain the increase in TBP production. Additionally, the specific metabolic pathways of TBP may account for the absence of a significant increasing trend in DBuP levels in humans.^36^ Previous studies have shown that DBuP is associated with thyroid dysfunction, insulin resistance, and preterm birth, emphasizing the need for long-term monitoring and further studies to identify these potential hazards.^37,38,39^

The results of our subgroup analysis highlight disparities in exposure levels among different sociodemographic groups. Children and youths generally exhibited higher concentrations of OPFR metabolites compared with adults, likely due to higher dust intake and hand-to-mouth behavior, which is consistent with previous studies.^40^ High exposure levels of OPFR metabolites among children and youths are a critical public health issue; therefore, further human biomonitoring of these compounds and their alternatives is needed. Additionally, there is growing evidence that education level, structural racism, individual and community poverty, regional policies, and residential environment are all associated with levels of pollutant exposure, which may partially explain the differences observed in our subgroups.^41,42^ Educational attainment may affect household income levels and consumer perceptions of health. Individuals with higher educational attainment are more likely to be aware of the health risks posed by OPFRs and to make informed choices to minimize exposure. Conversely, individuals with lower levels of educational attainment may be unaware of these risks, which may contribute to increased exposure. Labeling intended to inform consumers about hazardous substances has limited effects for individuals from lower socioeconomic backgrounds or with lower levels of educational attainment.^43^ These individuals may lack access to educational resources or may prioritize immediate economic needs over long-term health. Additionally, the technical language on labels can be a barrier to understanding risks. Obsolete products that do not meet new safety standards often circulate in secondhand markets or waste streams, disproportionately affecting low-income earners who seek affordable options.^44^ This highlights an important social justice issue, as economically disadvantaged groups face greater environmental health risks.

The findings of this study have substantial implications for both policy makers and public health. Disparities in exposure across different sociodemographic groups highlight a critical need for improvement. Ongoing efforts are needed to enhance the overall quality of OPFR preventive care. Ensuring that health care services and interventions for OPFR prevention are accessible and affordable for all individuals, regardless of socioeconomic status or education level, is essential.

### Limitations

This study has several limitations. First, the temporal variability of OPFR metabolites was not considered because urine samples were only measured once. Future studies should collect multiple urine samples to minimize the possibility of exposure misclassification. Additionally, the limited variety of OPFR metabolites and the lack of standardized protocols for clinical monitoring restricted our ability to assess the potential effect of regulation policies on OPFRs. Therefore, subsequent studies should focus on more OPFR exposures, especially emerging but understudied OPFRs, to establish standards for clinical OPFR metabolite monitoring and clarify the overall exposure patterns in the general US population. Third, NHANES response rates have decreased over time. However, we accounted for nonresponse bias by incorporating survey weights developed by the National Center for Health Statistics.

## Conclusions

In this cross-sectional study, urinary concentrations of OPFR metabolites in the US population exhibited varying trends from 2011 to 2020. We observed differences in exposure levels across subgroups in terms of sex, age, race and ethnicity, income, and education. Further research is required to explain the underlying causes of these disparities. Meanwhile, policies should consider socioeconomic factors to mitigate exposure and foster a more equitable society in which all individuals have the opportunity to live in a safe environment.

## Notes

Supplement 1. **eTable 1.** Trends in Organophosphate Flame-Retardant Concentrations by Population Subgroups Among Children and Youths, 2011**-**2020 NHANES **eTable 2.** Trends in Organophosphate Flame-Retardant Concentrations by Population Subgroups Among Adults, 2011**-**2020 NHANES **eFigure 1.** Geometric Mean of DPhP, BCPP, BCEtP, and DBuP Concentrations by Age Among US Children and Youths, 2011-2020 NHANES **eFigure 2.** Geometric Mean of DPhP, BCPP, BCEtP, and DBuP Concentrations by Sex Among US Children and Youths, 2011-2020 **eFigure 3.** Geometric Mean of DPhP, BCPP, BCEtP, and DBuP Concentrations by Race and Ethnicity Among US Children and Youths, 2011-2020 **eFigure 4.** Geometric Mean of DPhP, BCPP, BCEtP, and DBuP Concentrations by Parent Education Level Among US Children and Youths, 2011-2020 **eFigure 5.** Geometric Mean of DPhP, BCPP, BCEtP, and DBuP Concentrations by Household Income Among US Children and Youths, 2011-2020 **eFigure 6.** Geometric Mean of DPhP, BCPP, BCEtP, and DBuP Concentrations by Age Among US Adults, 2011-2020 **eFigure 7.** Geometric Mean of DPhP, BCPP, BCEtP, and DBuP Concentrations by Sex Among US Adults, 2011-2020 **eFigure 8.** Geometric Mean of DPhP, BCPP, BCEtP, and DBuP Concentrations by Race and Ethnicity Among US Adults, 2011-2020 **eFigure 9.** Geometric Mean of DPhP, BCPP, BCEtP, and DBuP Concentrations by Education Level Among US Adults, 2011-2020 **eFigure 10.** Geometric Mean of DPhP, BCPP, BCEtP, and DBuP Concentrations by Household Income Among US Adults, 2011-2020

Supplement 2.
Data Sharing Statement
