# Supplementary material for: Urinary Concentrations of Organophosphate Flame-Retardant Metabolites in the US Population
Source: JAMA Netw Open. 2024 Sep 25;7(9):e2435484. doi: 10.1001/jamanetworkopen.2024.35484 (PMC11425145; doi:10.1001/jamanetworkopen.2024.35484)
Supplement: Supplement 1. — eTable 1. Trends in Organophosphate Flame-Retardant Concentrations by Population Subgroups Among Children and Youths, 2011-2020 NHANES eTable 2. Trends in Organophosphate Flame-Retardant Concentrations by Population Subgroups Among Adults, 2011-2020 NHANES eFigure 1. Geometric Mean of DPhP, BCPP, BCEtP, and DBuP Concentrations by Age Among US Children and Youths, 2011-2020 NHANES eFigure 2. Geometric Mean of DPhP, BCPP, BCEtP, and DBuP Concentrations by Sex Among US Children and Youths, 2011-2020 eFigure 3. Geometric Mean of DPhP, BCPP, BCEtP, and DBuP Concentrations by Race and Ethnicity Among US Children and Youths, 2011-2020 eFigure 4. Geometric Mean of DPhP, BCPP, BCEtP, and DBuP Concentrations by Parent Education Level Among US Children and Youths, 2011-2020 eFigure 5. Geometric Mean of DPhP, BCPP, BCEtP, and DBuP Concentrations by Household Income Among US Children and Youths, 2011-2020 eFigure 6. Geometric Mean of DPhP, BCPP, BCEtP, and DBuP Concentrations by Age Among US Adults, 2011-2020 eFigure 7. Geometric Mean of DPhP, BCPP, BCEtP, and DBuP Concentrations by Sex Among US Adults, 2011-2020 eFigure 8. Geometric Mean of DPhP, BCPP, BCEtP, and DBuP Concentrations by Race and Ethnicity Among US Adults, 2011-2020 eFigure 9. Geometric Mean of DPhP, BCPP, BCEtP, and DBuP Concentrations by Education Level Among US Adults, 2011-2020 eFigure 10. Geometric Mean of DPhP, BCPP, BCEtP, and DBuP Concentrations by Household Income Among US Adults, 2011-2020 [file jamanetwopen-e2435484-s001.pdf]

# Supplementary Online Content

Huang YS, Shi HZ, Huang X, et al. Urinary concentrations of organophosphate flame-retardant metabolites in the US population. *JAMA Netw Open*. 2024;7(9):e2435484. doi:10.1001/jamanetworkopen.2024.35484

**eTable 1.** Trends in Organophosphate Flame-Retardant Concentrations by Population Subgroups Among Children and Youths, 2011-2020 NHANES

**eTable 2.** Trends in Organophosphate Flame-Retardant Concentrations by Population Subgroups Among Adults, 2011-2020 NHANES

**eFigure 1.** Geometric Mean of DPhP, BCPP, BCEtP, and DBuP Concentrations by Age Among US Children and Youths, 2011-2020 NHANES

**eFigure 2.** Geometric Mean of DPhP, BCPP, BCEtP, and DBuP Concentrations by Sex Among US Children and Youths, 2011-2020

**eFigure 3.** Geometric Mean of DPhP, BCPP, BCEtP, and DBuP Concentrations by Race and Ethnicity Among US Children and Youths, 2011-2020

**eFigure 4.** Geometric Mean of DPhP, BCPP, BCEtP, and DBuP Concentrations by Parent Education Level Among US Children and Youths, 2011-2020

**eFigure 5.** Geometric Mean of DPhP, BCPP, BCEtP, and DBuP Concentrations by Household Income Among US Children and Youths, 2011-2020

**eFigure 6.** Geometric Mean of DPhP, BCPP, BCEtP, and DBuP Concentrations by Age Among US Adults, 2011-2020

**eFigure 7.** Geometric Mean of DPhP, BCPP, BCEtP, and DBuP Concentrations by Sex Among US Adults, 2011-2020

**eFigure 8.** Geometric Mean of DPhP, BCPP, BCEtP, and DBuP Concentrations by Race and Ethnicity Among US Adults, 2011-2020

**eFigure 9.** Geometric Mean of DPhP, BCPP, BCEtP, and DBuP Concentrations by Education Level Among US Adults, 2011-2020

**eFigure 10.** Geometric Mean of DPhP, BCPP, BCEtP, and DBuP Concentrations by Household Income Among US Adults, 2011-2020

This supplementary material has been provided by the authors to give readers additional information about their work.

© 2024 Huang YS et al. *JAMA Network Open.*

**eTable 1.** Trends in Organophosphate Flame-Retardant Concentrations by Population Subgroups Among Children and Youths, 2011−2020 NHANES[a]

| | Children/Youths (6−19 years of age) | | | | $P_{trend}$[c] | $P_{int}$[d] |
|---|---|---|---|---|---|---|
| | **2011−2012** | **2013−2014** | **2015−2016** | **2017−2020** | | |
| | | | **DPhP** | | | |
| **Age group, y** | | | | | | |
| 6−12 | 1.54 (1.28−1.86) | 1.68 (1.41−2.00) | 2.09 (1.77−2.46) | 1.68 (1.50−1.88) | .32 | .23 |
| 13−19 | 1.57 (1.35−1.83) | 1.40 (1.17−1.67) | 1.84 (1.45−2.33) | 1.38 (1.22−1.56) | .44 | |
| **Sex** | | | | | | |
| Male | 1.13 (0.97−1.31) | 1.37 (1.14−1.63) | 1.85 (1.55−2.21) | 1.56 (1.39−1.76) | **<.001**[b] | **<.001** |
| Female | 2.18 (1.90−2.49) | 1.74 (1.50−2.01) | 2.08 (1.77−2.44) | 1.47 (1.30−1.65) | **<.001** | |
| **Race and ethnicity** | | | | | | |
| Hispanic | 1.51 (1.22−1.87) | 1.32 (1.02−1.69) | 2.00 (1.71−2.34) | 1.43 (1.26−1.63) | .82 | |
| Non-Hispanic | | | | | | .60 |
| Asian | 1.10 (0.80−1.53) | 1.17 (0.80−1.71) | 0.96 (0.73−1.27) | 0.99 (0.80−1.23) | .43 | |

© 2024 Huang YS et al. *JAMA Network Open.*

| | | | | | | |
|---|---|---|---|---|---|---|
| Black | 2.33 (1.82−2.99) | 1.85 (1.54−2.23) | 2.48 (1.99−3.08) | 1.90 (1.69−2.14) | .34 | |
| White | 1.43 (1.20−1.72) | 1.61 (1.38−1.89) | 1.89 (1.58−2.25) | 1.51 (1.34−1.71) | .57 | |
| Other[e] | 1.97 (1.10−3.53) | 1.43 (0.92−2.22) | 2.99 (1.93−4.65) | 1.60 (1.15−2.23) | .92 | |
| **Parental Educational level** | | | | | | |
| Some high school or lower | 1.54 (1.21−1.95) | 1.39 (1.07−1.80) | 1.90 (1.38−2.61) | NA | .31 | |
| High school graduate | 1.89 (1.62−2.19) | 1.68 (1.26−2.22) | 1.82 (1.35−2.45) | NA | .83 | |
| Some college | 1.94 (1.57−2.40) | 1.48 (1.17−1.88) | 2.11 (1.79−2.49) | NA | .39 | .29 |
| College graduate or above | 1.17 (0.89−1.53) | 1.58 (1.31−1.91) | 1.99 (1.66−2.39) | NA | **.003** | |
| **Ratio of family income to poverty level** | | | | | | |
| <1.3 | 1.78 (1.49−2.13) | 1.65 (1.31−2.08) | 1.94 (1.60−2.35) | 1.51 (1.24−1.85) | .38 | |
| 1.30−3.49 | 1.51 (1.35−1.69) | 1.40 (1.11−1.77) | 2.09 (1.74−2.51) | 1.56 (1.39−1.76) | .17 | .47 |
| ≥3.50 | 1.49 (1.16−1.90) | 1.53 (1.32−1.77) | 1.83 (1.46−2.31) | 1.50 (1.28−1.77) | .86 | |

© 2024 Huang YS et al. *JAMA Network Open.*

| | | | | | | |
|---|---|---|---|---|---|---|
| | | | **BCPP** | | | |
| **Age group, y** | | | | | | |
| 6−12 | 0.18 (0.16−0.21) | 0.26 (0.23−0.30) | 0.23 (0.18−0.30) | 0.16 (0.14−0.20) | .08 | .08 |
| 13−19 | 0.15 (0.13−0.17) | 0.18 (0.15−0.21) | 0.19 (0.14−0.24) | 0.15 (0.13−0.17) | .82 | |
| **Sex** | | | | | | |
| Male | 0.16 (0.15−0.18) | 0.21 (0.18−0.24) | 0.21 (0.17−0.27) | 0.17 (0.14−0.19) | .78 | .17 |
| Female | 0.17 (0.15−0.20) | 0.22 (0.19−0.26) | 0.20 (0.17−0.25) | 0.15 (0.12−0.18) | .11 | |
| **Race and ethnicity** | | | | | | |
| Hispanic | 0.18 (0.15−0.20) | 0.20 (0.17−0.23) | 0.20 (0.16−0.26) | 0.14 (0.12−0.16) | **0.008** | .26 |
| Non-Hispanic | | | | | | |
| Asian | 0.16 (0.12−0.21) | 0.24 (0.17−0.33) | 0.20 (0.15−0.26) | 0.17 (0.14−0.20) | .72 | |
| Black | 0.19 (0.16−0.22) | 0.21 (0.18−0.24) | 0.21 (0.16−0.28) | 0.15 (0.13−0.17) | **.03** | |
| White | 0.16 (0.14−0.18) | 0.22 (0.19−0.26) | 0.21 (0.16−0.27) | 0.17 (0.14−0.21) | .98 | |
| Other[e] | 0.16 (0.12−0.21) | 0.24 (0.17−0.32) | 0.29 (0.18−0.48) | 0.17 (0.13−0.21) | .60 | |

© 2024 Huang YS et al. *JAMA Network Open.*

| | | | | | | |
|---|---|---|---|---|---|---|
| **Parental Educational level** | | | | | | |
| Some high school or lower | 0.16 (0.13−0.20) | 0.17 (0.14−0.22) | 0.18 (0.14−0.24) | NA | .48 | |
| High school graduate | 0.17 (0.14−0.21) | 0.23 (0.17−0.29) | 0.16 (0.12−0.20) | NA | .52 | **.02** |
| Some college | 0.18 (0.15−0.22) | 0.21 (0.17−0.25) | 0.24 (0.18−0.32) | NA | .15 | |
| College graduate or above | 0.15 (0.13−0.17) | 0.25 (0.19−0.32) | 0.24 (0.18−0.33) | NA | **.007** | |
| **Ratio of family income to poverty level** | | | | | | |
| <1.3 | 0.16 (0.14−0.18) | 0.19 (0.16−0.23) | 0.19 (0.16−0.23) | 0.14 (0.11−0.17) | .19 | |
| 1.30−3.49 | 0.17 (0.14−0.20) | 0.21 (0.17−0.26) | 0.24 (0.18−0.32) | 0.16 (0.13−0.18) | .53 | .99 |
| ≥3.50 | 0.18 (0.13−0.24) | 0.26 (0.21−0.32) | 0.19 (0.14−0.26) | 0.18 (0.14−0.22) | .44 | |
| | | | **BCEtP** | | | |
| **Age group, y** | | | | | | |
| 6−12 | 0.71 (0.61−0.81) | 0.66 (0.55−0.79) | 0.64 (0.50−0.82) | 0.47 (0.40−0.55) | **<.001** | .28 |
| 13−19 | 0.66 (0.52−0.83) | 0.59 (0.50−0.70) | 0.63 (0.48−0.84) | 0.36 (0.31−0.41) | **<.001** | |

© 2024 Huang YS et al. *JAMA Network Open.*

| | | | | | | |
|---|---|---|---|---|---|---|
| **Sex** | | | | | | |
| Male | 0.64 (0.53−0.78) | 0.68 (0.58−0.78) | 0.63 (0.48−0.83) | 0.41 (0.35−0.48) | **<.001** | .73 |
| Female | 0.73 (0.60−0.88) | 0.57 (0.48−0.68) | 0.65 (0.53−0.79) | 0.40 (0.33−0.48) | **<.001** | |
| **Race and ethnicity** | | | | | | |
| Hispanic | 0.58 (0.48−0.72) | 0.70 (0.59−0.83) | 0.56 (0.46−0.68) | 0.37 (0.32−0.44) | **<.001** | |
| Non-Hispanic | | | | | | |
| Asian | 0.72 (0.50−1.03) | 0.65 (0.40−1.04) | 0.50 (0.33−0.75) | 0.44 (0.31−0.63) | .05 | .90 |
| Black | 0.78 (0.69−0.88) | 0.54 (0.49−0.60) | 0.67 (0.50−0.88) | 0.43 (0.37−0.51) | **<.001** | |
| White | 0.67 (0.53−0.85) | 0.61 (0.51−0.73) | 0.67 (0.51−0.86) | 0.39 (0.34−0.46) | **<.001** | |
| Other[e] | 1.19 (0.65−1.21) | 0.65 (0.42−1.02) | 0.95 (0.56−1.60) | 0.57 (0.42−0.78) | .12 | |
| **Parental Educational level** | | | | | | |
| Some high school or lower | 0.65 (0.53−0.81) | 0.60 (0.47−0.77) | 0.49 (0.40−0.59) | NA | .06 | |
| High school graduate | 0.83 (0.65−1.05) | 0.74 (0.52−1.03) | 0.63 (0.52−0.76) | NA | .09 | **.002** |
| Some college | 0.71 (0.51−0.99) | 0.60 (0.51−0.72) | 0.65 (0.49−0.86) | NA | .70 | |

© 2024 Huang YS et al. *JAMA Network Open.*

| | | | | | | |
|---|---|---|---|---|---|---|
| College graduate or above | 0.63 (0.47−0.84) | 0.59 (0.48−0.74) | 0.77 (0.56−1.06) | NA | .37 | |
| **Ratio of family income to poverty level** | | | | | | |
| <1.3 | 0.60 (0.50−0.72) | 0.55 (0.47−0.66) | 0.58 (0.46−0.72) | 0.40 (0.36−0.45) | **.001** | |
| 1.30−3.49 | 0.81 (0.66−0.98) | 0.76 (0.64−0.91) | 0.67 (0.52−0.86) | 0.48 (0.41−0.54) | **<.001** | .06 |
| ≥3.50 | 0.66 (0.46−0.94) | 0.58 (0.47−0.71) | 0.68 (0.47−1.00) | 0.39 (0.31−0.49) | **.01** | |
| | | | **DBuP** | | | |
| **Age group, y** | | | | | | |
| 6−12 | 0.14 (0.12−0.16) | 0.26 (0.22−0.32) | 0.16 (0.14−0.18) | 0.17 (0.15−0.20) | .83 | .71 |
| 13−19 | 0.10 (0.09−0.12) | 0.20 (0.17−0.24) | 0.12 (0.11−0.13) | 0.13 (0.12−0.15) | .49 | |
| **Sex** | | | | | | |
| Male | 0.11 (0.09−0.14) | 0.23 (0.20−0.27) | 0.13 (0.11−0.14) | 0.14 (0.13−0.15) | .72 | .32 |
| Female | 0.12 (0.11−0.14) | 0.23 (0.20−0.27) | 0.15 (0.13−0.16) | 0.17 (0.13−0.21) | .36 | |
| **Race and ethnicity** | | | | | | |

© 2024 Huang YS et al. *JAMA Network Open.*

| | | | | | | |
|---|---|---|---|---|---|---|
| Hispanic | 0.12 (0.10−0.14) | 0.22 (0.19−0.27) | 0.14 (0.12−0.15) | 0.17 (0.14−0.19) | .27 | |
| Non-Hispanic | | | | | | |
| Asian | 0.11 (0.087−0.13) | 0.14 (0.10−0.19) | 0.086 (0.079−0.093) | 0.12 (0.10−0.15) | .98 | .23 |
| Black | 0.14 (0.11−0.17) | 0.28 (0.24−0.33) | 0.15 (0.13−0.17) | 0.18 (0.16−0.20) | .80 | |
| White | 0.12 (0.099−0.14) | 0.23 (0.19−0.28) | 0.14 (0.13−0.15) | 0.14 (0.13−0.16) | .82 | |
| Other[e] | 0.11 (0.082−0.14) | 0.27 (0.19−0.38) | 0.13 (0.098−0.16) | 0.14 (0.12−0.17) | .49 | |
| **Parental Educational level** | | | | | | |
| Some high school or lower | 0.12 (0.099−0.14) | 0.23 (0.19−0.27) | 0.14 (0.13−0.16) | NA | **.03** | |
| High school graduate | 0.11 (0.088−0.13) | 0.23 (0.17−0.33) | 0.12 (0.10−0.15) | NA | .32 | .88 |
| Some college | 0.13 (0.11−0.16) | 0.21 (0.17−0.27) | 0.14 (0.12−0.17) | NA | .84 | |
| College graduate or above | 0.12 (0.095−0.16) | 0.25 (0.21−0.30) | 0.14 (0.12−0.16) | NA | .36 | |
| **Ratio of family income to poverty level** | | | | | | |
| <1.3 | 0.12 (0.11−0.14) | 0.25 (0.22−0.28) | 0.13 (0.12−0.14) | 0.16 (0.14−0.18) | .54 | .72 |

© 2024 Huang YS et al. *JAMA Network Open.*

| | | | | | |
|---|---|---|---|---|---|
| 1.30−3.49 | 0.11 (0.093−0.14) | 0.21 (0.17−0.27) | 0.15 (0.13−0.17) | 0.16 (0.14−0.18) | .12 |
| ≥3.50 | 0.13 (0.099−0.16) | 0.24 (0.19−0.30) | 0.13 (0.11−0.15) | 0.13 (0.11−0.15) | .13 |

Abbreviations: BCEtP: bis(2-chloroethyl) phosphate; BCPP: bis(1-chloro-2-propyl) phosphate; DBuP: dibutyl phosphate; DPhP: diphenyl phosphate; NA, not appliable; NHANES, National Health and Nutrition Examination Survey.

[a]: Organophosphate flame retardants levels were presented as geometric means due to the skewed distribution. Estimates were adjusted for NHANES survey weight and presented with 95% confidence intervals.

[b]: Significant P values have been emboldened.

[c]: The test for $P_{trend}$ across cycles evaluated the monotonic trend across the whole period.

[d]: P values for interaction.

[e]: Other includes American Indian or Alaska Native, Native Hawaiian or Other Pacific Islander, multiracial individuals, and those who do not identify as non-Hispanic White, non-Hispanic Black, non-Hispanic Asian, or Hispanic.

© 2024 Huang YS et al. *JAMA Network Open.*

**eTable 2.** Trends in Organophosphate Flame-Retardant Concentrations by Population Subgroups Among Adults, 2011−2020 NHANES[a]

| | Adults (≥20 years of age) | | | | $P_{trend}$[c] | $P_{int}$[d] |
|---|---|---|---|---|---|---|
| | **2011−2012** | **2013−2014** | **2015−2016** | **2017−2020** | | |
| | | | **DPhP** | | | |
| **Age group, y** | | | | | | |
| 20−39 | 1.06 (0.89−1.27) | 0.86 (0.75−0.98) | 1.01 (0.91−1.13) | 0.91 (0.81−1.03) | .37 | |
| 40−59 | 0.61 (0.51−0.74) | 0.64 (0.58−0.69) | 0.78 (0.68−0.90) | 0.65 (0.59−0.72) | .40 | **.02**[b] |
| ≥60 | 0.63 (0.52−0.76) | 0.64 (0.54−0.76) | 0.85 (0.72−1.02) | 0.70 (0.62−0.78) | .19 | |
| **Sex** | | | | | | |
| Male | 0.66 (0.59−0.74) | 0.66 (0.59−0.73) | 0.80 (0.70−0.92) | 0.72 (0.65−0.80) | .12 | .11 |
| Female | 0.85 (0.71−1.02) | 0.76 (0.69−0.83) | 0.96 (0.85−1.08) | 0.78 (0.72−0.84) | .64 | |
| **Race and ethnicity** | | | | | | |
| Hispanic | 0.78 (0.64−0.94) | 0.73 (0.64−0.84) | 0.87 (0.75−1.00) | 0.72 (0.64−0.81) | .68 | .57 |
| Non-Hispanic | | | | | | |

© 2024 Huang YS et al. *JAMA Network Open.*

| | | | | | | |
|---|---|---|---|---|---|---|
| Asian | 0.49 (0.39−0.62) | 0.46 (0.39−0.53) | 0.52 (0.40−0.67) | 0.41 (0.37−0.46) | .18 | |
| Black | 1.06 (0.93−1.21) | 0.92 (0.79−1.07) | 0.96 (0.73−1.26) | 0.87 (0.80−0.96) | .05 | |
| White | 0.74 (0.62−0.88) | 0.70 (0.63−0.77) | 0.92 (0.82−1.03) | 0.79 (0.70−0.89) | .23 | |
| Other[e] | 0.62 (0.35−1.11) | 0.62 (0.51−1.12) | 0.81 (0.59−1.09) | 0.65 (0.52−0.82) | .96 | |
| **Educational level** | | | | | | |
| Some high school or lower | 0.62 (0.49−0.79) | 0.77 (0.60−0.97) | 0.74 (0.65−0.84) | 0.69 (0.58−0.80) | .59 | .26 |
| High school graduate | 0.65 (0.49−0.85) | 0.69 (0.61−0.79) | 0.75 (0.66−0.86) | 0.81 (0.71−0.93) | .08 | |
| Some college | 0.96 (0.84−1.09) | 0.75 (0.68−0.82) | 0.97 (0.85−1.11) | 0.80 (0.71−0.90) | .28 | |
| College graduate or above | 0.72 (0.62−0.83) | 0.66 (0.58−0.75) | 0.94 (0.80−1.11) | 0.68 (0.58−0.79) | .94 | |
| **Ratio of family income to poverty level** | | | | | | |
| <1.3 | 0.85 (0.62−1.15) | 0.83 (0.75−0.92) | 0.94 (0.77−1.14) | 0.77 (0.67−0.89) | .70 | .75 |
| 1.30−3.49 | 0.73 (0.59−0.90) | 0.71 (0.63−0.80) | 0.91 (0.80−1.03) | 0.80 (0.73−0.88) | .20 | |

© 2024 Huang YS et al. *JAMA Network Open.*

| | | | | | | |
|---|---|---|---|---|---|---|
| ≥3.50 | 0.73 (0.60−0.88) | 0.65 (0.58−0.72) | 0.86 (0.77−0.96) | 0.72 (0.63−0.81) | .67 | |
| | | | **BCPP** | | | |
| **Age group, y** | | | | | | |
| 20−39 | 0.17 (0.14−0.20) | 0.22 (0.19−0.24) | 0.19 (0.16−0.23) | 0.13 (0.12−0.15) | **.002** | |
| 40−59 | 0.14 (0.13−0.16) | 0.16 (0.14−0.18) | 0.17 (0.14−0.22) | 0.13 (0.12−0.15) | .36 | .09 |
| ≥60 | 0.14 (0.12−0.16) | 0.16 (0.14−0.18) | 0.18 (0.15−0.22) | 0.13 (0.12−0.14) | .11 | |
| **Sex** | | | | | | |
| Male | 0.15 (0.13−0.17) | 0.19 (0.17−0.21) | 0.20 (0.16−0.24) | 0.14 (0.13−0.15) | .06 | .30 |
| Female | 0.15 (0.14−0.17) | 0.17 (0.15−0.18) | 0.17 (0.14−0.20) | 0.13 (0.12−0.13) | **<.001** | |
| **Race and ethnicity** | | | | | | |
| Hispanic | 0.15 (0.14−0.17) | 0.17 (0.15−0.19) | 0.18 (0.16−0.21) | 0.13 (0.12−0.14) | **0.009** | |
| Non-Hispanic | | | | | | .69 |
| Asian | 0.17 (0.14−0.21) | 0.18 (0.14−0.23) | 0.17 (0.11−0.26) | 0.13 (0.11−0.15) | **.02** | |
| Black | 0.18 (0.16−0.20) | 0.18 (0.16−0.20) | 0.18 (0.15−0.21) | 0.13 (0.12−0.15) | **<.001** | |

© 2024 Huang YS et al. *JAMA Network Open.*

| | | | | | | |
|---|---|---|---|---|---|---|
| White | 0.15 (0.13−0.17) | 0.18 (0.16−0.20) | 0.18 (0.14−0.23) | 0.13 (0.12−0.14) | **.03** | |
| Other[e] | 0.12 (0.084−0.18) | 0.22 (0.15−0.34) | 0.23 (0.14−0.37) | 0.12 (0.10−0.15) | .54 | |
| **Educational level** | | | | | | |
| Some high school or lower | 0.14 (0.12−0.17) | 0.16 (0.14−0.19) | 0.16 (0.13−0.21) | 0.13 (0.12−0.14) | .31 | |
| High school graduate | 0.15 (0.13−0.18) | 0.16 (0.12−0.17) | 0.20 (0.16−0.25) | 0.13 (0.12−0.15) | .23 | .15 |
| Some college | 0.15 (0.13−0.18) | 0.18 (0.16−0.21) | 0.19 (0.15−0.23) | 0.13 (0.12−0.14) | .08 | |
| College graduate or above | 0.16 (0.13−0.19) | 0.21 (0.18−0.24) | 0.18 (0.14−0.22) | 0.13 (0.12−0.14) | **.007** | |
| **Ratio of family income to poverty level** | | | | | | |
| <1.3 | 0.15 (0.13−0.18) | 0.17 (0.14−0.19) | 0.19 (0.15−0.23) | 0.12 (0.11−0.14) | .07 | |
| 1.30−3.49 | 0.15 (0.13−0.17) | 0.17 (0.15−0.18) | 0.19 (0.15−0.23) | 0.13 (0.12−0.15) | .24 | .25 |
| ≥3.50 | 0.16 (0.14−0.18) | 0.20 (0.17−0.22) | 0.18 (0.14−0.22) | 0.14 (0.13−0.15) | **.01** | |
| **BCEtP** | | | | | | |

© 2024 Huang YS et al. *JAMA Network Open.*

| | | | | | | |
|---|---|---|---|---|---|---|
| **Age group, y** | | | | | | |
| 20−39 | 0.49 (0.39−0.62) | 0.43 (0.37−0.51) | 0.47 (0.39−0.57) | 0.31 (0.26−0.37) | **.002** | |
| 40−59 | 0.45 (0.35−0.59) | 0.35 (0.31−0.39) | 0.35 (0.29−0.43) | 0.29 (0.25−0.34) | **.005** | .11 |
| ⩾60 | 0.34 (0.28−0.40) | 0.33 (0.28−0.40) | 0.40 (0.31−0.52) | 0.28 (0.24−0.32) | .10 | |
| **Sex** | | | | | | |
| Male | 0.47 (0.42−0.53) | 0.41 (0.35−0.48) | 0.44 (0.36−0.55) | 0.30 (0.26−0.34) | **<.001** | .33 |
| Female | 0.40 (0.32−0.51) | 0.34 (0.30−0.39) | 0.37 (0.32−0.44) | 0.29 (0.25−0.33) | **.01** | |
| **Race and ethnicity** | | | | | | |
| Hispanic | 0.50 (0.40−0.64) | 0.42 (0.35−0.51) | 0.40 (0.35−0.45) | 0.27 (0.23−0.31) | **<.001** | |
| Non-Hispanic | | | | | | |
| Asian | 0.44 (0.36−0.54) | 0.31 (0.28−0.34) | 0.29 (0.22−0.39) | 0.22 (0.18−0.27) | **<.001** | .88 |
| Black | 0.70 (0.63−0.79) | 0.46 (0.41−0.52) | 0.45 (0.38−0.52) | 0.34 (0.27−0.41) | **<.001** | |
| White | 0.39 (0.32−0.46) | 0.35 (0.32−0.39) | 0.40 (0.31−0.51) | 0.30 (0.27−0.34) | **.04** | |
| Other[e] | 0.43 (0.27−0.70) | 0.43 (0.25−0.76) | 0.67 (0.50−0.90) | 0.31 (0.20−0.49) | .36 | |

© 2024 Huang YS et al. *JAMA Network Open.*

| | | | | | | |
|---|---|---|---|---|---|---|
| **Educational level** | | | | | | |
| Some high school or lower | 0.46 (0.39−0.54) | 0.39 (0.33−0.45) | 0.42 (0.36−0.49) | 0.27 (0.23−0.32) | **<.001** | .73 |
| High school graduate | 0.41 (0.32−0.53) | 0.35 (0.30−0.41) | 0.41 (0.32−0.51) | 0.32 (0.29−0.36) | .07 | |
| Some college | 0.49 (0.39−0.63) | 0.44 (0.36−0.55) | 0.49 (0.38−0.63) | 0.31 (0.26−0.36) | **.002** | |
| College graduate or above | 0.38 (0.31−0.46) | 0.31 (0.26−0.37) | 0.33 (0.26−0.41) | 0.27 (0.23−0.32) | **.02** | |
| **Ratio of family income to poverty level** | | | | | | |
| <1.3 | 0.49 (0.37−0.64) | 0.48 (0.41−0.57) | 0.46 (0.39−0.55) | 0.31 (0.27−0.36) | **.005** | |
| 1.30−3.49 | 0.45 (0.38−0.53) | 0.38 (0.32−0.45) | 0.50 (0.40−0.62) | 0.31 (0.27−0.36) | **.009** | .47 |
| ≥3.50 | 0.38 (0.30−0.48) | 0.31 (0.28−0.35) | 0.33 (0.27−0.41) | 0.27 (0.23−0.31) | **.02** | |
| | | | **DBuP** | | | |
| **Age group, y** | | | | | | |
| 20−40 | 0.11 (0.098−0.12) | 0.17 (0.15−0.21) | 0.11 (0.10−0.11) | 0.13 (0.12−0.14) | .87 | .94 |

© 2024 Huang YS et al. *JAMA Network Open.*

| | | | | | | |
|---|---|---|---|---|---|---|
| 40−60 | 0.11 (0.10−0.12) | 0.16 (0.14−0.19) | 0.099 (0.092−0.11) | 0.13 (0.11−0.14) | .74 | |
| >60 | 0.12 (0.11−0.13) | 0.20 (0.17−0.23) | 0.11 (0.098−0.12) | 0.15 (0.13−0.16) | .82 | |
| **Sex** | | | | | | |
| Male | 0.11 (0.10−0.12) | 0.18 (0.15−0.21) | 0.10 (0.10−0.11) | 0.12 (0.11−0.13) | .09 | **.001** |
| Female | 0.11 (0.11−0.12) | 0.17 (0.15−0.20) | 0.10 (0.098−0.11) | 0.15 (0.14−0.16) | .08 | |
| **Race and ethnicity** | | | | | | |
| Hispanic | 0.11 (0.090−0.13) | 0.17 (0.14−0.21) | 0.11 (0.10−0.12) | 0.13 (0.12−0.14) | .77 | |
| Non-Hispanic | | | | | | |
| Asian | 0.094 (0.085−0.10) | 0.10 (0.087−0.13) | 0.088 (0.083−0.093) | 0.10 (0.096−0.11) | .44 | .42 |
| Black | 0.12 (0.10−0.13) | 0.21 (0.18−0.25) | 0.11 (0.10−0.12) | 0.14 (0.13−0.16) | .81 | |
| White | 0.12 (0.11−0.12) | 0.18 (0.15−0.21) | 0.10 (0.097−0.11) | 0.14 (0.12−0.15) | .91 | |
| Other[e] | 0.089 (0.077−0.10) | 0.15 (0.098−0.22) | 0.14 (0.12−0.16) | 0.13 (0.096−0.17) | .16 | |
| **Educational level** | | | | | | |
| Some high school or lower | 0.11 (0.10−0.12) | 0.18 (0.15−0.22) | 0.12 (0.10−0.13) | 0.14 (0.12−0.15) | .34 | **.04** |

© 2024 Huang YS et al. *JAMA Network Open.*

| | | | | | | |
|---|---|---|---|---|---|---|
| High school graduate | 0.11 (0.10−0.11) | 0.17 (0.14−0.20) | 0.10 (0.092−0.11) | 0.15 (0.13−0.17) | .05 | |
| Some college | 0.12 (0.10−0.13) | 0.19 (0.16−0.22) | 0.11 (0.10−0.12) | 0.13 (0.12−0.14) | .41 | |
| College graduate or above | 0.11 (0.097−0.13) | 0.17 (0.14−0.20) | 0.098 (0.090−0.11) | 0.12 (0.11−0.13) | .34 | |
| **Ratio of family income to poverty level** | | | | | | |
| <1.3 | 0.11 (0.099−0.12) | 0.18 (0.16−0.21) | 0.11 (0.10−0.12) | 0.14 (0.13−0.15) | .19 | |
| 1.30−3.49 | 0.11 (0.10−0.12) | 0.17 (0.14−0.20) | 0.11 (0.10−0.11) | 0.14 (0.13−0.16) | .18 | **.04** |
| ≥3.50 | 0.11 (0.10−0.13) | 0.18 (0.15−0.21) | 0.098 (0.093−0.10) | 0.12 (0.12−0.13) | .15 | |

Abbreviations: BCEtP: bis(2-chloroethyl) phosphate; BCPP: bis(1-chloro-2-propyl) phosphate; DBuP: dibutyl phosphate; DPhP: diphenyl phosphate; NA, not appliable; NHANES, National Health and Nutrition Examination Survey.

[a]: Organophosphate flame retardants levels were presented as geometric means due to the skewed distribution. Estimates were adjusted for NHANES survey weight and presented with 95% confidence intervals.

[b]: Significant P values have been emboldened.

[c]: The test for $P_{trend}$ across cycles evaluated the monotonic trend across the whole period.

[d]: P values for interaction.

[e]: Other includes American Indian or Alaska Native, Native Hawaiian or Other Pacific Islander, multiracial individuals, and those who do not identify as non-Hispanic White, non-Hispanic Black, non-Hispanic Asian, or Hispanic.

© 2024 Huang YS et al. *JAMA Network Open.*

**eFigure 1.** Geometric Mean of DPhP, BCPP, BCEtP, and DBuP Concentrations by Age Among US Children and Youths, 2011-2020

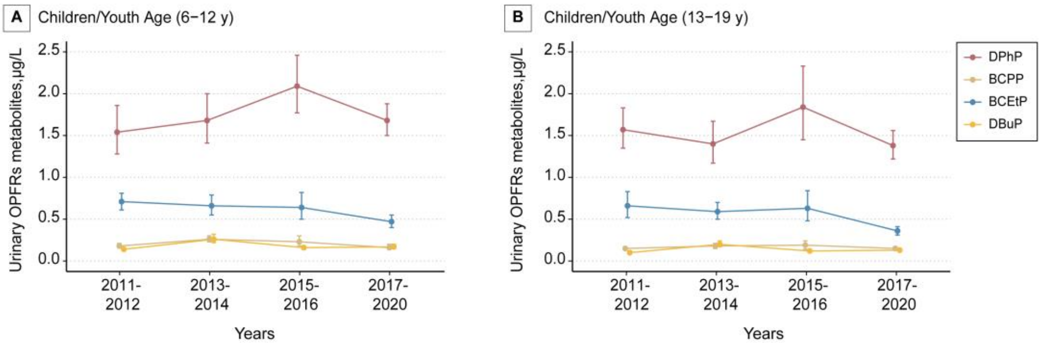


A, Trends in OPFRs metabolites concentrations among U.S. children/youths aged 6−12 years. Data are presented incorporating sample weights and are adjusted for clusters and strata of the complex sample design of the National Health and Nutrition Examination Survey, 2011 to 2020. B, Trends in OPFRs metabolites concentrations among U.S. children/youths aged 13−19 years.

© 2024 Huang YS et al. *JAMA Network Open.*

**eFigure 2.** Geometric Mean of DPhP, BCPP, BCEtP, and DBuP Concentrations by Sex Among US Children and Youths, 2011-2020

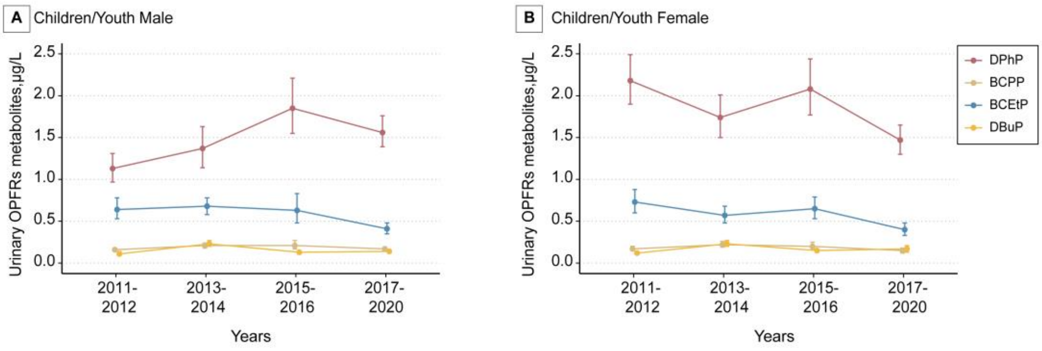


A, Trends in OPFRs metabolites concentrations among male U.S. children/youths. Data are presented incorporating sample weights and are adjusted for clusters and strata of the complex sample design of the National Health and Nutrition Examination Survey, 2011 to 2020. B, Trends in OPFRs metabolites concentrations among female U.S. children/youths.

© 2024 Huang YS et al. *JAMA Network Open.*

**eFigure 3.** Geometric Mean of DPhP, BCPP, BCEtP, and DBuP Concentrations by Race and Ethnicity Among US Children and Youths, 2011-2020

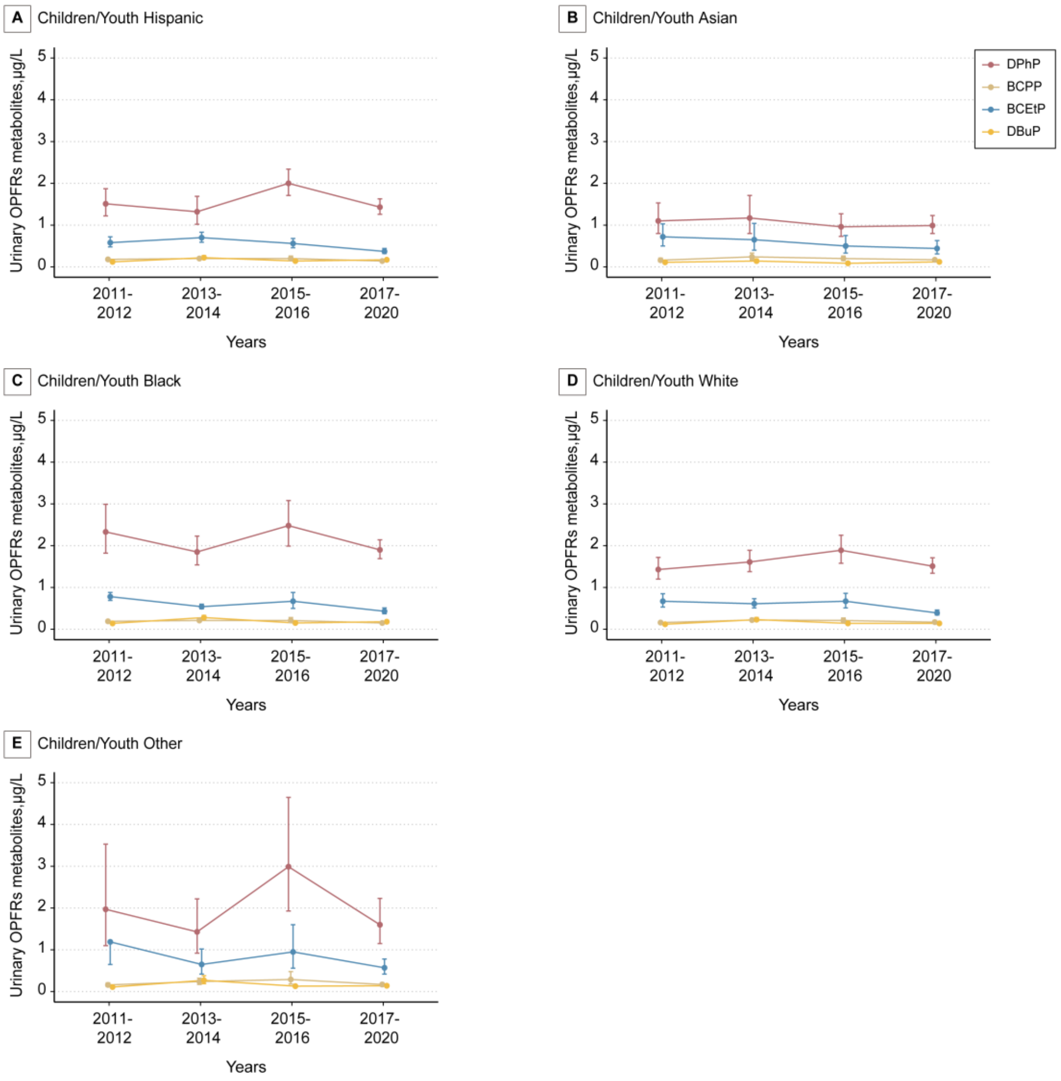


A, Trends in OPFRs metabolites concentrations among U.S. children/youths of Hispanic race and ethnicity. Data are presented incorporating sample weights and are adjusted for clusters and strata of the complex sample design of the National Health and Nutrition Examination Survey, 2011 to 2020. B, Trends in OPFRs metabolites concentrations among U.S. children/youths of Asian race and ethnicity. C, Trends in OPFRs metabolites concentrations among U.S. children/youths of Black race and ethnicity. D, Trends in OPFRs metabolites concentrations among U.S. children/youths of White race and ethnicity. E, Trends in OPFRs metabolites concentrations among U.S. children/youths of other races and ethnicities.

© 2024 Huang YS et al. *JAMA Network Open.*

**eFigure 4.** Geometric Mean of DPhP, BCPP, BCEtP, and DBuP Concentrations by Parent Education Level Among US Children and Youths, 2011-2020

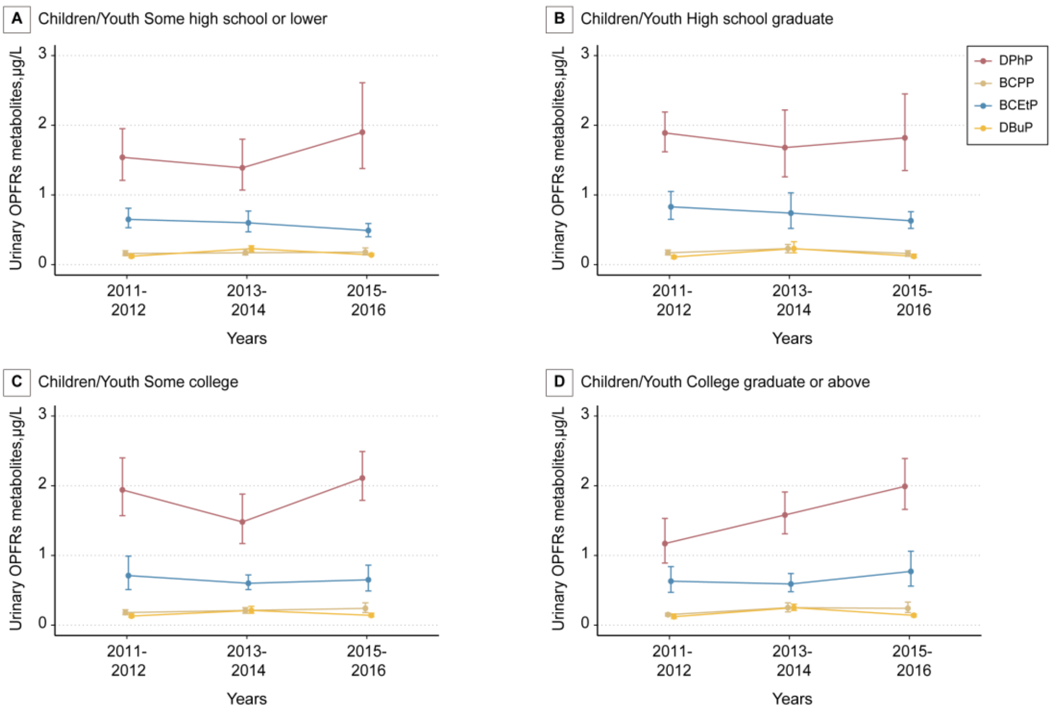


A, Trends in OPFRs metabolites concentrations among U.S. children/youths whose parents have some high school education or lower. Data are presented incorporating sample weights and are adjusted for clusters and strata of the complex sample design of the National Health and Nutrition Examination Survey, 2011 to 2020. B, Trends in OPFRs metabolites concentrations among U.S. children/youths whose parents graduated from high school. C, Trends in OPFRs metabolites concentrations among U.S. children/youths whose parents have some college education. D, Trends in OPFRs metabolites concentrations among U.S. children/youths whose parents are college graduates or have higher education.

© 2024 Huang YS et al. *JAMA Network Open.*

**eFigure 5.** Geometric Mean of DPhP, BCPP, BCEtP, and DBuP Concentrations by Household Income Among US Children and Youths, 2011-2020

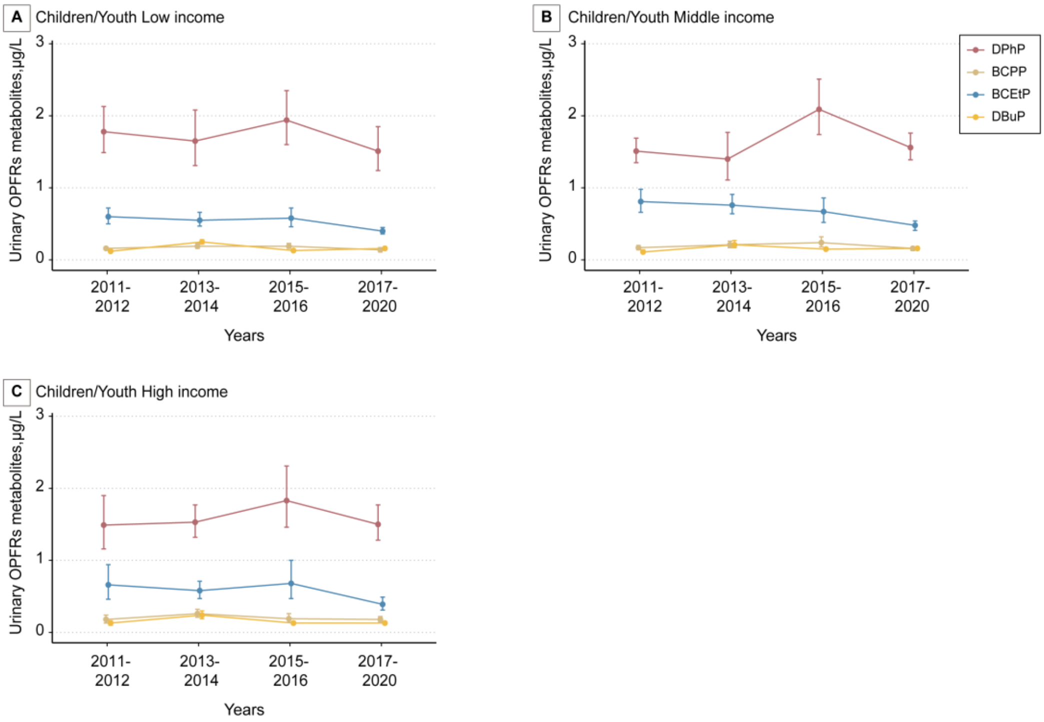


A, Trends in OPFRs metabolites concentrations among U.S. children/youths from low-income households. Data are presented incorporating sample weights and are adjusted for clusters and strata of the complex sample design of the National Health and Nutrition Examination Survey, 2011 to 2020. B, Trends in OPFRs metabolites concentrations among U.S. children/youths from middle-income households. C, Trends in OPFRs metabolites concentrations among U.S. children/youths from high-income households.

© 2024 Huang YS et al. *JAMA Network Open.*

**eFigure 6.** Geometric Mean of DPhP, BCPP, BCEtP, and DBuP Concentrations by Age Among US Adults, 2011-2020

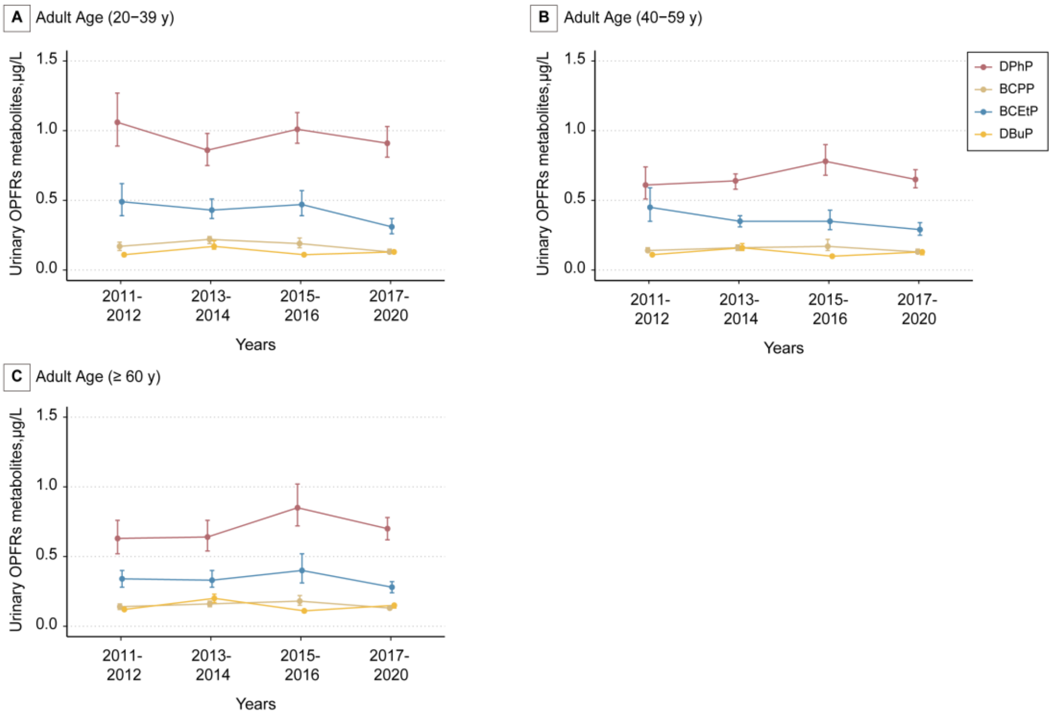


A, Trends in OPFRs metabolites concentrations among U.S. adults aged 20−39 years. Data are presented incorporating sample weights and are adjusted for clusters and strata of the complex sample design of the National Health and Nutrition Examination Survey, 2011 to 2020. B, Trends in OPFRs metabolites concentrations among U.S. adults aged 40−59 years. C, Trends in OPFRs metabolites concentrations among U.S. adults aged 60 years or older.

© 2024 Huang YS et al. *JAMA Network Open.*

**eFigure 7.** Geometric Mean of DPhP, BCPP, BCEtP, and DBuP Concentrations by Sex Among US Adults, 2011-2020

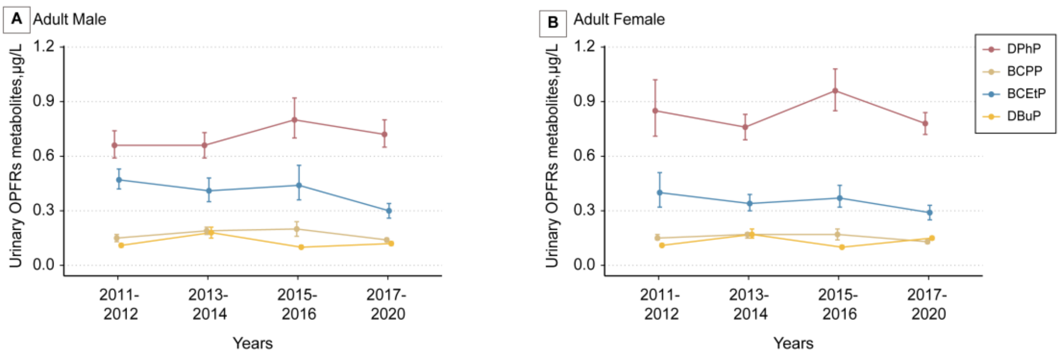


A, Trends in OPFRs metabolites concentrations among male U.S. adults. Data are presented incorporating sample weights and are adjusted for clusters and strata of the complex sample design of the National Health and Nutrition Examination Survey, 2011 to 2020. B, Trends in OPFRs metabolites concentrations among female U.S. adults.

© 2024 Huang YS et al. *JAMA Network Open.*

**eFigure 8.** Geometric Mean of DPhP, BCPP, BCEtP, and DBuP Concentrations by Race and Ethnicity Among US Adults, 2011-2020

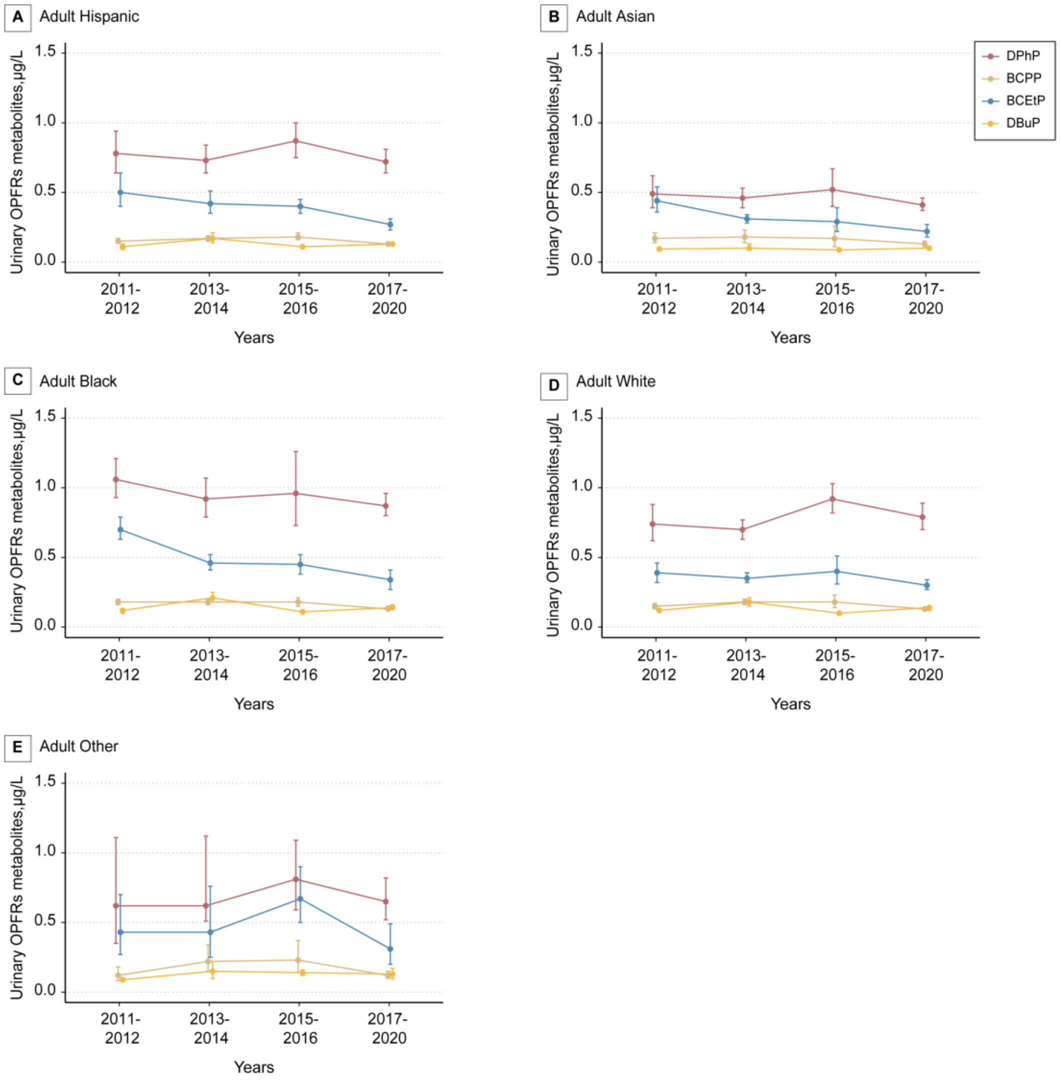


A, Trends in OPFRs metabolites concentrations among U.S. adults of Hispanic race and ethnicity. Data are presented incorporating sample weights and are adjusted for clusters and strata of the complex sample design of the National Health and Nutrition Examination Survey, 2011 to 2020. B, Trends in OPFRs metabolites concentrations among U.S. adults of Asian race and ethnicity. C, Trends in OPFRs metabolites concentrations among U.S. adults of Black race and ethnicity. D, Trends in OPFRs metabolites concentrations among U.S. adults of White race and ethnicity. E, Trends in OPFRs metabolites concentrations among U.S. adults of other races and ethnicities.

© 2024 Huang YS et al. *JAMA Network Open.*

**eFigure 9.** Geometric Mean of DPhP, BCPP, BCEtP, and DBuP Concentrations by Education Level Among US Adults, 2011-2020

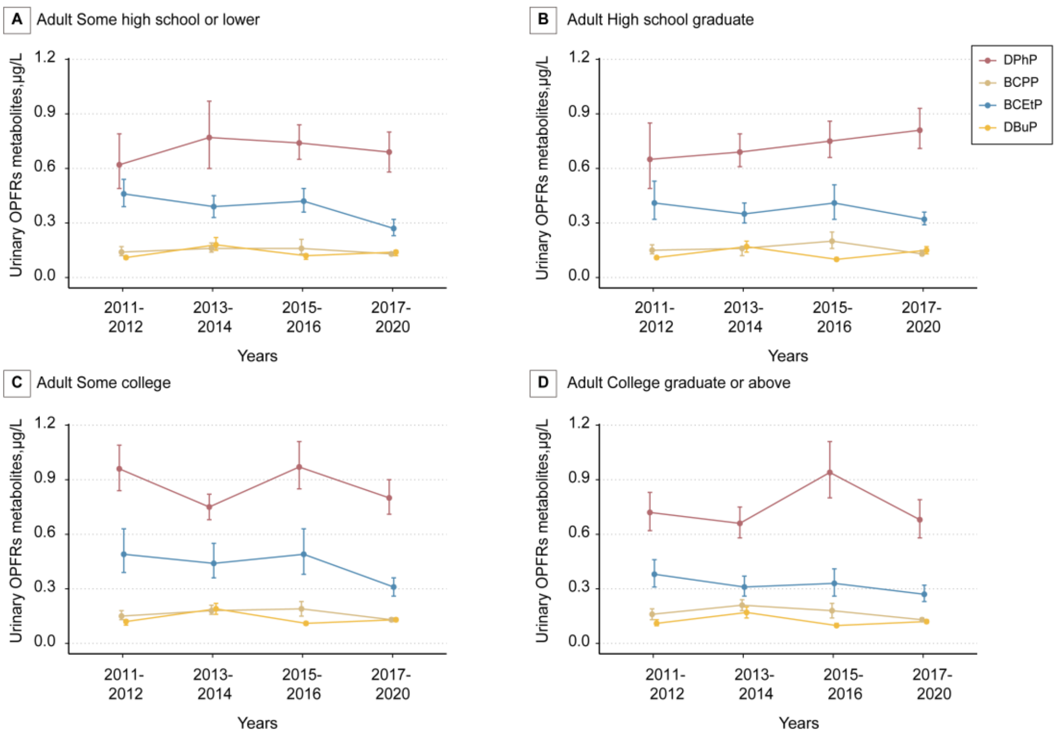


A, Trends in OPFRs metabolites concentrations among U.S. adults who have some high school education or lower. Data are presented incorporating sample weights and are adjusted for clusters and strata of the complex sample design of the National Health and Nutrition Examination Survey, 2011 to 2020. B, Trends in OPFRs metabolites concentrations among U.S. adults who graduated from high school. C, Trends in OPFRs metabolites concentrations among U.S. adults who have some college education. D, Trends in OPFRs metabolites concentrations among U.S. adults who are college graduates or have higher education.

© 2024 Huang YS et al. *JAMA Network Open.*

**eFigure 10.** Geometric Mean of DPhP, BCPP, BCEtP, and DBuP Concentrations by Household Income Among US Adults, 2011-2020

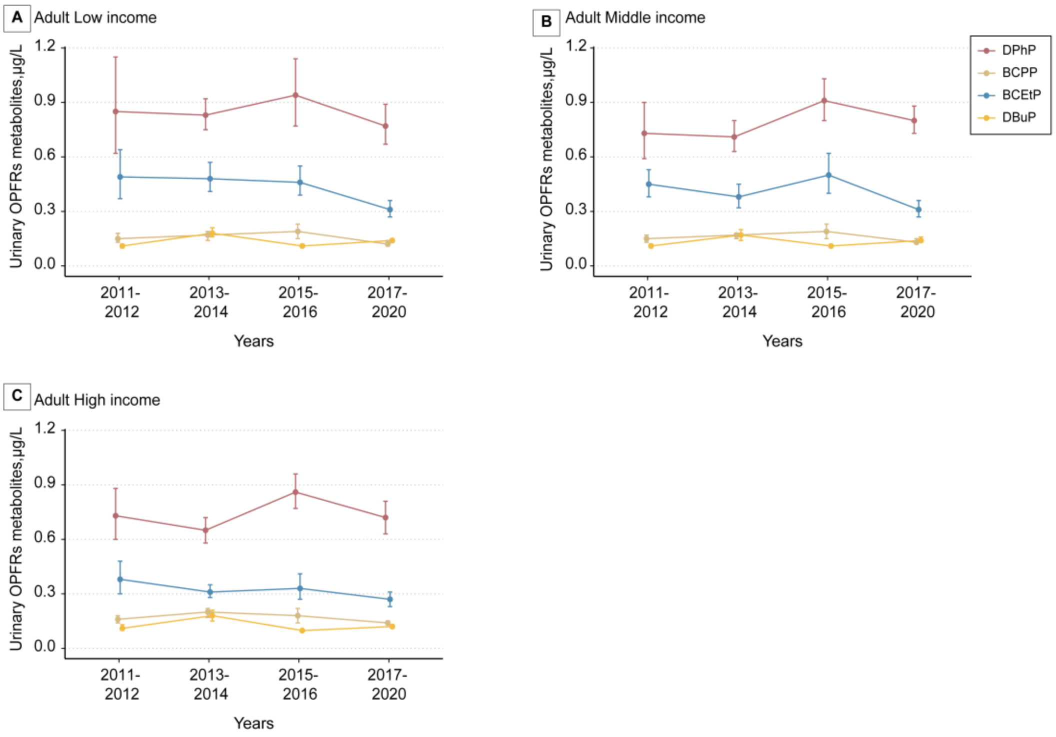


A, Trends in OPFRs metabolites concentrations among U.S. adults from low-income households. Data are presented incorporating sample weights and are adjusted for clusters and strata of the complex sample design of the National Health and Nutrition Examination Survey, 2011 to 2020. B, Trends in OPFRs metabolites concentrations among U.S. adults from middle-income households. C, Trends in OPFRs metabolites concentrations among U.S. adults from high-income households.

© 2024 Huang YS et al. *JAMA Network Open.*
