# Supplementary material for: Urinary Concentrations of Organophosphate Flame-Retardant Metabolites in the US Population
Source: JAMA Netw Open. 2024 Sep 25;7(9):e2435484. doi: 10.1001/jamanetworkopen.2024.35484 (PMC11425145; doi:10.1001/jamanetworkopen.2024.35484)
Supplement: Supplement 2. — Data Sharing Statement [file jamanetwopen-e2435484-s002.pdf]

Data Sharing Statement

Huang. Urinary Concentrations of Organophosphate Flame-Retardant Metabolites in the US Population. *JAMA Netw Open*. Published September 25, 2024.
doi:10.1001/jamanetworkopen.2024.35484

Data
Data available: No
